# Editorial: Fatigue in multiple sclerosis—A current perspective

**DOI:** 10.3389/fneur.2023.1150717

**Published:** 2023-02-07

**Authors:** Anna Pokryszko-Dragan, Iris-Katharina Penner, Giancarlo Comi

**Affiliations:** ^1^Department and Clinic of Neurology, Wroclaw Medical University, Wroclaw, Poland; ^2^Department of Neurology and Neurorehabilitation, Inselspital, University Hospital Bern, Bern, Switzerland; ^3^MS Centre Casa di Cura Igea, Vita Salute San Raffaele University, Milan, Italy

**Keywords:** multiple sclerosis, fatigue, fatigability, pathophysiology, neuroimaging, monitoring, depression

As editors of this Research Topic, we wanted to acknowledge and give scientific space to a symptom that affects so many patients with multiple sclerosis (MS) and which has an enormous impact on the daily lives of those affected, their vocational status, and social participation. It may also affect their adherence to disease-modifying therapies, interfering with expected treatment outcomes (1). Despite recent progress in our understanding of the background of MS, as well as the availability of therapeutic options, understanding the pathophysiology and management of fatigue still remains a challenge (2–4). Thus, there is a need for better recognition of this problem, based on focused research with further clinical implications.

The present Research Topic aims to highlight the current view on risk factors and mechanisms of fatigue in MS, as well as its assessment and management throughout the disease.

The review article by Patejdl and Zettl focuses on the pathophysiology of motor fatigue and fatigability. It gives a comprehensive overview of current concepts, including definitions, assessment approaches, pathophysiology, and training interventions.

Ayache et al. reappraise neurophysiological studies in view of putative mechanisms of fatigue and fatigability in MS. Among the parameters of CNS excitability, evaluated with the use of transcranial magnetic stimulation, those associated with movement preparation and facilitation seem the most consistently related to fatigue. Furthermore, the therapeutic potential of non-invasive brain stimulation is discussed for the short- and long-term amelioration of motor and cognitive fatigability, considering innovative protocols and their combination with pharmacotherapy or exercise.

The first original article by Broscheid, Behrens, Bilgin-Egner, et al. is focused on the meaningfulness of gait parameters in the context of motor performance fatigability (PF) on the one hand and the relevance of minimum toe clearance (MTC) in the quantification of motor PF in people with MS (pwMS) on the other. Importantly, based on the 6-min walk test (6MWT) it was discovered that the second minute of the test delivered more stable gait parameters than the first minute and that MTC in combination with other spatiotemporal gait parameters was not able to detect motor PF, although, there was a decrease in MTC variability observed in some pwMS toward the end of the 6MWT. These results indicate the weakness of reliable data acquisition during the first minute of the 6MWT and also point to the necessity of longer test intervals to discover motor PF.

In the second original article by Broscheid, Behrens, Dettmers, et al., the 6MWT is combined with a cognitive task to create a motor-cognitive dual-task performance to simulate multi-tasking behavior in a real-life setting. PwMS and healthy controls (HCs) had to perform a fast version of the 6MWT, while at the same time performing an arithmetic task. At the same time, the hemodynamic response of their prefrontal cortex was recorded. The results showed an effect on cognitive PF but not on motor PF although participants reported being physically fatigued. The PFC activation remained unchanged. Again, the authors suggest that the 6MWT is, even in the fast version, not long enough to induce objectifiable motor PF.

Tarasiuk et al. in their review on the co-occurrence of fatigue and depression in MS, highlight pathomechanisms potentially shared by these conditions. They include proinflammatory cytokine response and oxidative/nitrosative stress which affect the tryptophane metabolic pathway, impairment of the hypothalamic-pituitary-adrenal axis, and disturbed functionality of cortico-subcortical loop (prefrontal cortex, basal ganglia, and limbic system). Psychosocial aspects of fatigue and depression, their reciprocal relationships, and the need for differentiation are also discussed.

Links between fatigue and mental health in MS are addressed in the cross-sectional study by AlSaeed et al. In the study group of pwMS with mild disability, almost half reported fatigue, and up to 26% presented with symptoms of anxiety or depression. Fatigue level was found to correlate significantly with depression, anxiety, and quality of life, with no relationship between fatigue and demographics or physical activity.

There are two modalities to assess fatigue: asking patients with questionnaires and measuring the impact of fatigue on physical and cognitive functions (fatigability). Block et al., in their review, highlight the value and limitations of the two approaches. In principle, fatigue is a subjective experience, so it has to be explored with self-reported questionnaires, however, this active patient-reported outcome has the problem of recall bias and does not inform about the day-to-day variability of the symptom. On the other hand, the evaluation of physical and cognitive decline with neurophysiological and psychometric tests has the advantage of the objectivity of the measures, however, they may not reflect the subjective perception of fatigue. The authors emphasize the value of remote monitoring with smartphones and wearable devices because they provide a more granular collection of both the patient-reported state and quantify physical and cognitive performances. Block et al. conclude that the combination of fatigue and fatigability measures using remote monitoring may provide a more comprehensive outcome in clinical and research settings.

The problem of the variability of fatigue over time is also addressed by Grothe et al., who examine the month-to-month changes in the perceived level of motor and cognitive fatigue. In a retrospective monocentric cohort study, they find that fatigue was lower during winter and higher during summer, with a nadir in August. However, the oscillations of the fatigue score were modest. Fatigue levels correlated with monthly temperature. The authors underline the importance of taking these seasonal changes in fatigue into consideration in interventional studies on fatigue because they may influence the results.

Many studies have approached the problem of brain magnetic resonance correlates of fatigue (5, 6), and most of them have shown the important role in the involvement of the striato-thalamo-cortical network. The Román et al. study, using an advanced diffusion imaging technique, examines the correlations of white matter and basal ganglia microstructure measures with the rate of cognitive fatigue over time during a fatigue-inducing task. Patients with cognitive fatigability had more severe damage to white matter tracts associated with basal ganglia connectivity, confirming the key role of the fatigue network.

These articles in the Research Topic contribute to shedding light on the most mysterious symptom of the disease - fatigue, which is so difficult to measure because of its multidimensionality and so difficult to treat. In a recent survey of the PROMS initiative, jointly promoted by the International Federation of MS and European Charcot Foundation, pwMS pointed out that fatigue is the first daily problem they have to face and they expressed the importance to improve outcome measures of fatigue with a fundamental patient contribution.

## Author contributions

AP-D, I-KP, and GC equally contributed to conception and design of the article, writing particular sections, and revision of the whole text. All authors contributed to the article and approved the submitted version.

## References

[1] Oliva Ramirez A Keenan A Kalau O Worthington E Cohen L Singh S. Prevalence and burden of multiple sclerosis-related fatigue: a systematic literature review. BMC Neurol. (2021) 21:468. 10.1186/s12883-021-02396-1 34856949 PMC8638268

[2] Palotai M Guttmann CR. Brain anatomical correlates of fatigue in multiple sclerosis. Mult Scler. (2020) 26:751–64. 10.1177/1352458519876032 31536461

[3] Chalah MA Kauv P Créange A Hodel J Lefaucheur JP Ayache SS. Neurophysiological, radiological and neuropsychological evaluation of fatigue in multiple sclerosis. Mult Scler Relat Disord. (2019) 28:145–52. 10.1016/j.msard.2018.12.029 30594815

[4] Nourbakhsh B Revirajan N Morris B Cordano C Creasman J Manguinao M. Safety and efficacy of amantadine, modafinil, and methylphenidate for fatigue in multiple sclerosis: a randomised, placebo-controlled, crossover, double-blind trial. Lancet Neurol. (2021) 20:38–48. 10.1016/S1474-4422(20)30354-9 33242419 PMC7772747

[5] Nakagawa S Takeuchi H Taki Y Nouchi R Kotozaki Y Shinada T.. Basal ganglia correlates of fatigue in young adults. Sci Rep. (2016) 6:1–7. 10.1038/srep21386 26893077 PMC4759547

[6] Jameen ARM Ribbons K Lechner-Scott J Ramadan S. Evaluation of MS related central fatigue using MR neuroimaging methods: scoping review. J Neurol Sci. (2019) 400:52–71. 10.1016/j.jns.2019.03.007 30903860

